# VR-SOAP, a modular virtual reality treatment for improving social activities and participation of young people with psychosis: a study protocol for a single-blind multi-centre randomized controlled trial

**DOI:** 10.1186/s13063-023-07241-z

**Published:** 2023-04-15

**Authors:** Ivo Alexander Meins, Dauw Catharina Muijsson-Bouwman, Saskia Anne Nijman, Kirstin Greaves-Lord, Wim Veling, Gerdina Hendrika Maria Pijnenborg, Elisabeth Christine Dorothée van der Stouwe

**Affiliations:** 1 University Center of Psychiatry, University Medical Center Groningen, University of Groningen, Groningen, Netherlands; 2 Department of Clinical and Developmental Neuropsychology, University of Groningen, Groningen, Netherlands; 3 GGZ Drenthe, Langdurige Zorg, Assen, Netherlands; 4 Jonx Groningen, Groningen, Netherlands

**Keywords:** Social functioning, Psychotic disorder, Virtual Reality, Personalized Treatment

## Abstract

**Background:**

Young people with a psychotic disorder have the same social goals as their healthy peers, but their social networks are smaller, they participate less often in leisure activities and are less successful in work and education. Causes of these problems are multifaceted, but culminate in difficulties with interacting in daily life social situations. Current treatments have only moderate effects on social functioning and often target one specific domain. Virtual reality (VR) has the potential to improve the treatment of social interaction difficulties. We developed a modular VR treatment for social functioning and participation (VR-SOAP). In this study, the effect of this intervention will be investigated in a randomized controlled trial (RCT).

**Methods:**

A total of 116 participants (age 18–40) with a DSM-5 diagnosis of schizophrenia spectrum or other psychotic disorder and problems with social functioning will be recruited from mental healthcare institutes in the Netherlands. Participants will be randomized to the experimental condition (VR-SOAP) or active VR control condition (VRelax). VR-SOAP consists of 14 sessions and 5 modules addressing causes of impaired social functioning: four optional modules (1–4) and one fixed module (5). Vrelax consists of 14 sessions that entail psychoeducation, stress management, relaxation techniques, and the exploration of relaxing environments in VR. Primary outcomes are quantity and quality of social contacts, leisure activities and social participation, measured with the experience sampling method (ESM). Secondary outcomes are psychiatric symptoms, social behaviour, social cognition, self-esteem, self-stigma and paranoid thoughts. Treatment effects will be compared at pre-treatment (baseline), post-treatment and at 6-month follow-up.

**Discussion:**

If VR-SOAP proves to be effective, it provides therapists with a much-needed tool to improve social functioning of young adults with a psychotic disorder. Additionally, since the treatment consists of multiple modules targeting different transdiagnostic factors, this trial might provide input for new treatments to improve social functioning in a range of symptoms and disorders, e.g. mood, autism spectrum and anxiety disorders.

**Trial registration:**

On the 10th of November 2021, this trial was registered prospectively in the Dutch Trial Register as NL9784.

## Background

Young people with a psychotic disorder have the same social goals as their peers [1, 2], but their social networks are smaller, they participate less often in social leisure activities and they are less successful in their work and educational pursuits [2–5]. A reduction in the quantity and quality of social contacts, leisure activities, and participation in society are core aspects of impaired social functioning. In turn, reduced social functioning among young people with psychosis is associated with a lower quality of life [2], depressive symptoms and social anxiety symptoms [6]. Estimates of a good functional outcome after a first episode of psychosis range between 17 and 45% [7, 8]. Moreover, 20 years after their first episode of psychosis, the majority of patients still have impaired social functioning [9]. Since social functioning impairments have a major and persistent impact on a patient’s quality of life and come with a large societal cost [10], effective treatment is vital.

In recent decades, several interventions have been developed to improve the social functioning of patients with a psychotic disorder, e.g. social skills training, social cognition training and cognitive behavioural therapy. However, these approaches have shown only moderate effects on social functioning [11–13]. As they generally target a single aspect, they may not sufficiently capture the multifaceted nature of social functioning problems in psychosis.

Based on the literature, we identified the main determinants of impaired social functioning in patients with recent onset psychosis [14, 15]. For example, negative symptoms, in particular impaired motivation and pleasure, are a barrier to successful engagement in social activities [16–18]. Additionally, social cognition problems are strongly related to impaired social functioning [19]. Furthermore, paranoid ideations and social anxiety can result in avoidance and safety behaviours, complicating social functioning [20, 21]. Likewise, low self-esteem and self-stigma make social interactions challenging [22]. Importantly, patients often have reduced social skills [14, 23]. These causes jointly culminate in difficulties interacting in daily life social situations. Therefore, any treatment targeting social functioning problems should identify and address an individual’s array of multiple overlapping and interrelated causes.

Recently, there has been an interest in developing personalized interventions. Among these personalized interventions are modular treatments. Modular treatments consist of several interconnected, yet independent modules [24]. A literature review of interventions for individuals at risk for psychosis emphasized the need for modular treatments for young people with psychosis, as these treatments could effectively meet their varying individual needs [25]. Preliminary results of a recently developed modular treatment for persecutory delusions in psychosis were promising: patients were satisfied with the treatment and believed that it helped [26].

Another challenge in traditional treatments for social functioning is generalizability, as it is difficult for patients to implement the skills acquired during therapy into their daily lives [23, 27]. Skills should be trained in an environment similar to everyday life [27]. However, this can be time-consuming and is not always possible in a conventional clinical setting. Virtual reality (VR) offers this possibility and has great potential to improve the training of social functioning among patients with psychosis [28–31]. With VR, patients can repeatedly practice social interactions in a simulated three-dimensional real-life setting. Using VR, real-life emotional and behavioural responses can be evoked and behaviour can be practiced in a safe and controlled setting [23, 28]. Preliminary studies have indicated that VR treatment for social skills among individuals with psychosis is feasible and may be effective [23, 32].

To address both the multifaceted nature of each individual’s social functioning problems and the concern of generalizability, our lab in collaboration with persons with lived experience, developed a Virtual Reality Treatment for Social Activities and Participation (VR-SOAP); a personalized, modular VR treatment to promote social activities and participation among young people (18–40 years) with psychosis. VR-SOAP consists of various optional modules that target several processes that are thought to underlie impaired social functioning [33].

This paper outlines the study protocol of a randomized controlled trial to investigate whether VR-SOAP is superior to an active control condition (Vrelax). The primary aim of the study is to test whether VR-SOAP improves the quantity and quality of social contacts, leisure activities and social participation using the Experience Sampling Method (ESM). Second, the study will investigate whether the effects of VR-SOAP are mediated by changes in the contributing causes of impaired social functioning (i.e. negative symptoms, social cognition problems, paranoid ideations, low self-esteem, self-stigma, and social skills).

## Methods/design

Ethics approval for the study was obtained from The Medical Ethics Review Board of the University Medical Center Groningen (number NL69628.042.21). The study is registered in the Netherlands Trial Register (number: NL9784).

### Participants/setting

To reach the desired sample size, participants will be recruited from multiple mental healthcare centres in the Netherlands (University Center of Psychiatry of the University Medical Center Groningen, GGZ Drenthe, Dimence, Mentrum, KieN-VIP, Altrecht and GGZ Rivierduinen). Patients receiving ambulatory care will be included. Eligible and interested participants will be referred by their clinician and contacted by researchers.

Inclusion criteria are:

- □ DSM-5 [34] diagnosis of schizophrenia spectrum or other psychotic disorder determined by a semi-structured diagnostic interview.
- □ Reduced quantity or quality of social contacts, leisure activities or social participation, according to the treating clinician or patient.
- □ Age 18–40 years.

Exclusion criteria are:

- □ IQ < 70 estimated by treating clinician.
- □ Insufficient command of the Dutch language.
- □ Photosensitive epilepsy.

If the diagnosis of a schizophrenia spectrum or other psychotic disorder has not been established according to DSM-5 criteria with a semi-structured interview in the past 5 years, the Mini-SCAN [35] will be administered to confirm clinical diagnosis. The interview will target psychotic episodes over a lifetime period.

Participants can receive a maximum of 40 euros for the three measurements and will be reimbursed for any travel expenses during the assessment.

### Design

**Fig. 1:**
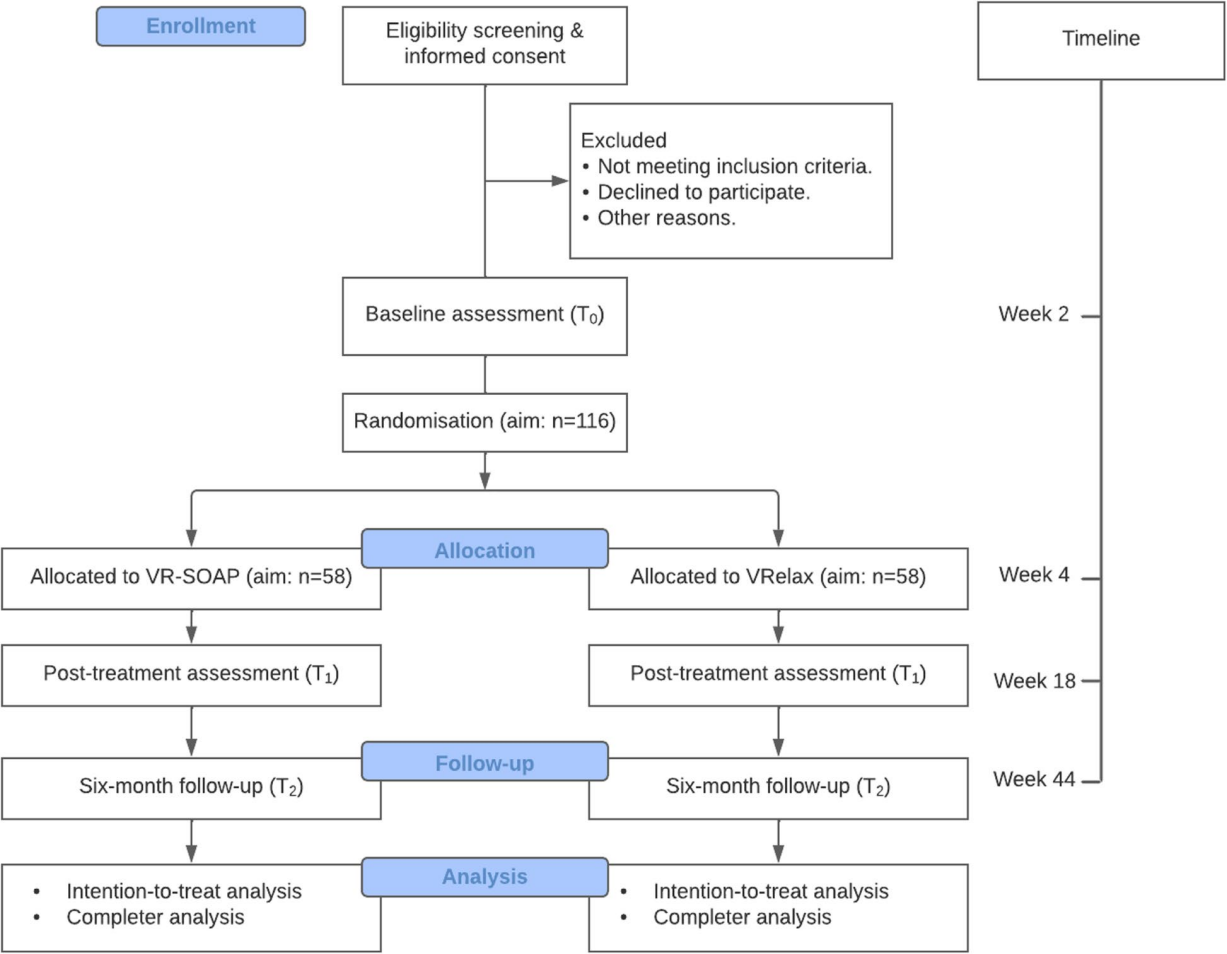
Flowchart of study

See Fig. 1 for the CONSORT flow diagram. The study is a multi-centre single-blind randomized controlled trial (RCT) with two conditions: the experimental VR treatment (VR-SOAP) and VR relaxation (VRelax) as an active control condition.

Both groups continue to receive treatment as usual (TAU) during the study, which can include antipsychotic medication and contacts with a psychiatric nurse and/or psychiatrist. Both groups participate in an assessment before the start of treatment (T0), at post-treatment (T1) and 6 months after the end of treatment (T2).

### Interventions

#### VR-SOAP (experimental condition)

VR-SOAP is a VR treatment of 14 individual weekly sessions of 60 min with a psychologist. The sessions are provided with a detailed treatment protocol and session forms to ensure treatment integrity. Therapists who hold at least a master’s degree in clinical psychology and a basic qualification to provide cognitive behavioural therapy (CBT) deliver VR-SOAP.

**Fig. 2:**
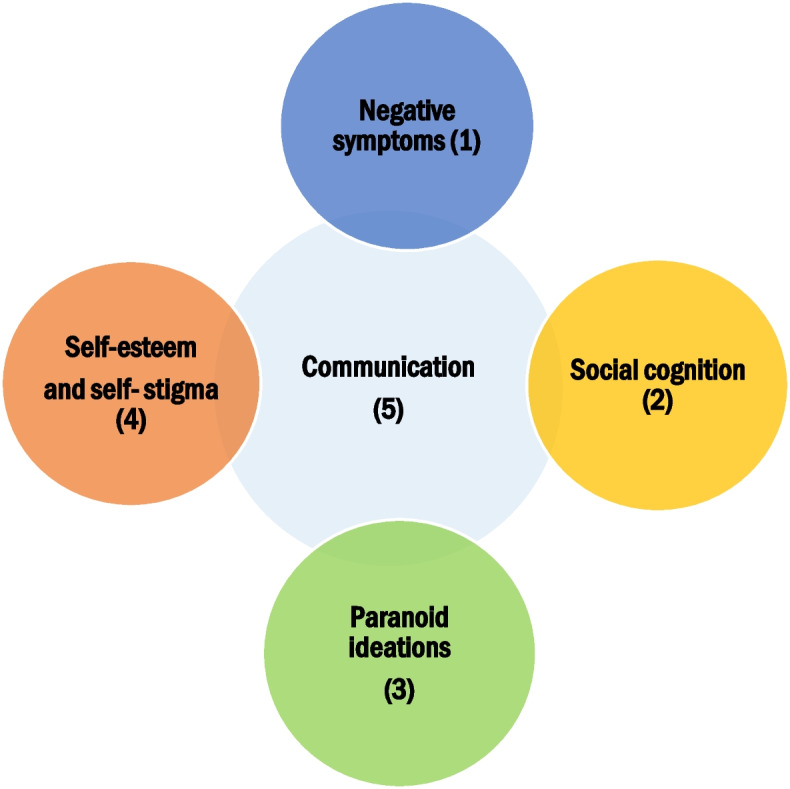
The modules of VR-SOAP

VR-SOAP consists of five modules (Fig. 2) that address the identified various causes of impaired social functioning: four optional modules (1–4) and one fixed module (5). In sessions 1–2, the patient and therapist discuss the baseline assessment (T0) and formulate goals concerning social contacts, leisure activities and/or social participation. After session 2, the patient and therapist select two of the optional VR modules (4 sessions each), depending on a combination of the perceived causes of the patient’s social interaction difficulties, the results of the baseline assessment and the patient’s goals:

- Module 1: Domain—Negative symptoms; Treatment components—behavioural activation and planning in social situations, demoralization, training attention control and information processing.
- Module 2: Domain—Social cognition; Treatment components—recognizing facial emotions, interpreting social situations, mentalizing.
- Module 3: Domain—Paranoid ideations and social anxiety; Treatment components—behavioural experiments testing harm expectancies, exposure exercises, dropping safety behaviour.
- Module 4: Domain—Self-esteem and self-stigma; Treatment components—improving positive self-image in interactions, practicing disclosure of mental illness.

All participants will end with the Communication and Interaction skills module (4 sessions), in which experiences, knowledge and skills from the other modules are integrated and applied.

- Module 5: Domain—Communication and Interaction skills; Treatment components—practicing conversations, job interviews, attending a party—with a focus on social skills, e.g. responding and sending, affiliative, instrumental role and interactional skills.

##### Session 3–14

At least half of every session is spent in VR. Exercises and role-plays are guided by the therapist and evaluated afterwards, using cognitive-behavioural techniques. They can be adapted and repeated during the session. At the end of each session, homework is given, aimed at applying the session’s insights and obtained skills in daily life, with exercises designed for achieving the patient’s social goals. These exercises will be followed up at the beginning of the next session.

VR-SOAP consists of animated, interactive virtual social environments, programmed by CleVR B.V. in Unity3D software. The virtual social environments that are used for social interactions include the following: a café, shopping street, office, living room, supermarket, park and bus. Characteristics of social situations can be customized, such as the number, gender and ethnic appearance of the virtual characters (avatars) present in the social situation. Additionally, the behaviour of avatars (gestures, facial emotion expression and speech) can be controlled in real time. Participants wear an Oculus Rift head-mounted display with an HD resolution of 2160 × 1200 and a field of view of 110°, and navigate through the virtual environments using a controller. Therapists speak with the patient (via a microphone with voice distortion) as an avatar and can control the movement and gestures of avatars.

#### VRelax (active control condition)

The control condition also consists of 14 individual weekly VR sessions (60 min) with a therapist, but aims at relaxation, using the VRelax app, programmed by VRelax B.V [36]. Participants wear the Oculus Quest 2 head-mounted display and watch 360° videos with interactive game elements. Multiple natural environments are available, e.g. swimming with dolphins, a beach and a forest. Audio clips of guided relaxation exercises such as progressive muscle relaxation and meditation are embedded in the videos. Participants can navigate between environments, activate relaxation exercises and use game elements by looking at hotspots within the videos. Similar to VR-SOAP, the sessions are provided using a detailed treatment protocol and both therapists and participants use a workbook. The VRelax therapy is carried out by therapists with at least a bachelor’s degree in psychology or professionals with clinical experience with the patient population.

In session 1, the participant and therapist identify the main sources of stress for the participant. All remaining VRelax sessions consist of practicing stress management techniques (e.g. grounding or body scanning), psychoeducation (e.g. the effect of stress on mental health) and VR relaxation. Half of each session is allocated to relaxation in VR.

VRelax is selected as the active control condition to control for non-specific treatment effects of VR interventions. VRelax does not target underlying causes of impaired social functioning. In line with this, it has been shown that VRelax does not have an effect on social functioning [37].

### Measures

Table 1 provides an overview of the measures used in this study.

**Table 1:** Measures used in this study

Domain	Instrument	Method	Baseline assessment (T0)	Post-treatment assessment (T1)	Follow-up assessment (T2)
**Primary outcome measures**					
Social Functioning in daily life	Diary protocol mapping social interactions, activities and momentary mental states	Experience Sampling Method (ESM)	x	x	x
**Secondary outcome measures**					
Social functioning	Social Functioning Scale (SFS)	Questionnaire	x	x	x
Interpersonal behaviour	Inventory Interpersonal Situations (SIG)	Questionnaire	x	x	x
Self-esteem	Self-Esteem Rating Scale Short-Form (SERS-SF)	Questionnaire	x	x	x
Social Interaction Anxiety	Social Interaction Anxiety Scale (SIAS)	Questionnaire	x	x	x
Stigma	Internalized Stigma of Mental Illness Scale (ISMI)	Questionnaire	x	x	x
Dating and assertion	Dating and Assertion Questionnaire (DAQ)	Questionnaire	x	x	x
Paranoid Thoughts	Revised Green et al. Persecutory and Paranoid Thought Scale (R- GPTS)	Questionnaire	x	x	x
Theory of Mind	Hinting task	Task	x	x	x
Emotion Recognition	Bell-Lysaker Emotion Recognition Task (BLERT)	Task	x	x	x
Negative Symptoms	Brief Negative Symptom Scale (BNSS)	Semi-structured Interview	x	x	x
Psychotic symptoms	Positive and Negative Syndrome Scale (PANSS)	Semi-structured Interview	x	x	x
**Other measures**					
Presence	I-group Presence Questionnaire (IPQ)	Questionnaire		x	
Demographic and clinical background	Questions	Questionnaire	x	x	x
Diagnostic	Mini-SCAN	Semi-structured Interview	x		
Therapeutic Alliance	Session Rating Form	Questionnaire	S1- S14		

#### Primary outcome

##### Quantity and quality of social contacts, leisure activities and social participation

Three domains of social functioning are measured with the experience sampling method (ESM). Participants will complete ESM diaries at semi-random moments, prompted by a signal on their phone, five times a day for 2 weeks. Items are scored on a Likert scale ranging from 0 (not at all) to 7 (very) or as multiple-choice questions.

The diary consists of 35 items and will take 2–3 min to complete. Each time the participant is asked whether he or she is or has engaged in any (social) activity, at that particular moment, and since the previous questionnaire. If the participant is engaged in a (social) activity, the participant is asked additional questions, e.g. I feel accepted by this company. The diary parallels the five modules such that different underlying processes targeted in VR-SOAP are assessed (e.g. we expect the item: “I find this activity pleasurable”, to be associated with negative symptoms.).

#### Secondary outcomes

##### Social functioning

- Social functioning will be assessed using the Social Functioning Scale (SFS). The SFS is developed for patients with schizophrenia and measures specifically those areas crucial for community functioning [38]. Additionally, the SFS distinguishes between a lack of competence and a lack of performance [38]. The SFS consists of 78 questions with varying response formats. The total score of the SFS is the mean of the seven subdomains; social engagement/withdrawal; interpersonal behaviour; pro-social activities; recreation; independence-competence; independence-performance; employment/occupation [39]. The SFS is self-administered separately by the patient and a relative or friend. The results of both will be reported.

##### Determinants of impaired social functioning

The identified determinants of impaired social functioning and subsequent domains addressed in the five modules of the VR-SOAP interventions were admitted as secondary outcomes.

- *Negative symptoms*: The Brief Negative Symptom Scale examines blunted affect, alogia, asociality, anhedonia and avolition [40]. The BNSS consists of 13 items rated from normal to very severe (6-point) and is administered as a semi-structured interview. Item scores are based on the week before the interview.
- *Social cognition*: Social cognition will be assessed by means of two tasks. The Hinting Task assesses the capacity to infer the true meaning behind indirect speech [41]. The Hinting Task depicts ten short social situations in which a hint is dropped. Each narrative and question is read aloud by the test leader. The Dutch version of the Bell and Lysaker emotion recognition task (BLERT) consists of 35 videos in which actors narrate a situation and express an emotion or remain neutral [42]. Emotions that can be expressed are as follows: happiness, sadness, fear, anger, disgust and surprise. After each video, the patient is asked to choose the correct emotion out of the seven options.
- *Paranoid ideation*: The Revised Green Persecutory Thoughts Scale is a self-report questionnaire consisting of 8 items measuring ideas of reference and 10 items measuring ideas of prosecution [43]. Both subscales will be analysed separately.
- *Social anxiety*: The Social Interaction Anxiety Scale (SIAS) is a self-report questionnaire of 19 items measuring fear of social interaction [44]
- *Self-esteem*: The Self-esteem Rating Scale Short-form is a twenty-item self-report questionnaire measuring positive and negative self-esteem separately [43, 45]
- *Self-stigma*: The Internalized Stigma of Mental Illness scale assesses stigma with 29 items on five different subscales; Alienation, Stereotype Endorsement, Discrimination Experience, Social Withdrawal, Stigma Resistance [46].
- *Social behaviour*: Social behaviour (e.g. social interaction and assertiveness) will be measured by means of two assessment instruments. The Inventory of Interpersonal situations (ISS) measures an interactive concept of social anxiety and avoidance using 50 items describing hypothetical responses to social situations that the patient will score on two dimensions; frequency and discomfort [47]. The Dating and Assertion Questionnaire is a self-report questionnaire that consists of 18 items to identify appropriate dating and assertiveness behaviours [41, 48]. The first 9 items inquire the likelihood of engaging in dating and assertiveness behaviours, whereas the latter 9 items probe the degree of discomfort in doing so or incompetence otherwise [41].

##### Other

- Psychotic symptoms will be assessed by means of the Positive and Negative Syndrome Scale (PANSS). The PANSS is a semi-structured interview that assesses positive symptoms, negative symptoms and general psychopathology [49]. The PANSS consists of 30 items pertaining to the week prior to the interview and is scored from normal to very severe (7-point).
- To measure presence, defined as “the sense of being in the virtual environment” [50] the I-group Presence Questionnaire [51] is administered once at post-treatment. The IPQ is a self-report questionnaire of 14 items.

##### During sessions

To measure therapeutic alliance, the patient fills in the Session Rating Form (SRS) in the therapist workbook at the end of each session.

### Study Procedures

#### Recruitment

Patients who might be eligible for participation will be contacted by their treating clinician (psychiatrist, doctor, psychologist, nursing specialist). The treating clinician informs the patient about the study. If the patient is interested and agrees to share contact details, the patient will be screened and informed by the researchers, both written and verbal. Additionally, the clinician will be contacted by the researcher to determine whether impairments are present in the following domains: work and education, (social) leisure activities or social contacts. Subsequently, the patient will receive written information and is given 1 week to consider participation. If the patient agrees to participate, the informed consent will be signed by the participant and the research assistant. The baseline assessment (T0) will be conducted afterwards. After randomization, a summary of the baseline assessment is provided to the patient and the therapist to guide the selection of VR-SOAP modules.

#### Assessment

Trained independent research assistants blinded to treatment allocation will do the post-treatment and follow-up assessments. Blinding is achieved by instructing therapists and participants to not communicate group allocation to assessors. To evaluate blindness, the assessor fills in the self-report form. A sensitivity analysis of data acquired by blinded assessors only will be done to investigate the effect of blinding.

Participants who are completing the ESM will be contacted at least twice during the data collection period to ensure a high response rate, with additional follow-ups if the response rate falls below 33%.

#### Randomization

With the open-source “Research Randomizer” (randomizer.org), a list is created for each participating centre in which each participant number is assigned either to VR-SOAP (experimental condition) or VRelax (active control condition). Randomization is 1:1, there are no randomization blocks. To guarantee concealment of treatment allocation, the bureau of randomization assigns each participant after baseline assessment to a participant number in order of inclusion and communicates the allocation to the researcher.

Baseline assessments are repeated in the week after the last treatment (T1) and 6 months after the end of treatment (T2).

#### Protocol fidelity

Therapists complete a training, which amounts to 3 days for VR-SOAP therapists and 1 day for VRelax therapists. This difference is due to the extensive CBT and modular structure of VR-SOAP. Additionally, therapists will attend monthly supervision in which all ongoing cases will be discussed to ensure the quality and fidelity of both treatment conditions. Supervision will be provided by a trained (VR) therapist who is part of the research group. Every therapist fills in a session form after each session to register protocol fidelity and anomalies.

Deviations in the research protocol will be communicated to METc, the sponsor and participating centres and registered in the Dutch Trial Register.

### Statistical analyses

All analyses will be intention to treat. The intention-to-treat analysis will include all participants who have given their informed consent and have been randomized to a group after the baseline assessment. Descriptive statistics will be calculated for all clinical and outcome variables as well as for sociodemographic variables. Means that differ significantly at baseline between groups will be added as a covariate to the analysis. For each domain of the primary outcome variable, mean total scores are calculated based on all 70 scores (5 times a day × 14 days = 70 scores). Multilevel mixed-model regression analysis will be used for the analysis of the primary outcome (ESM). Repeated assessments will be nested within participants and all models will include a random intercept for participant. The method of estimation is maximum likelihood and the covariance structure independent. Treatment effects are evaluated with group (VR-SOAP or VRelax) × time (baseline, end of treatment, follow-up) interaction terms. Measures taken after treatment and follow-up will be evaluated separately.

A mediator analysis will be conducted to identify the underlying processes of change in the primary outcome. The primary analysis will be followed up with sensitivity analysis on completers, i.e. patients who completed 12 or more sessions. In case of missing data, participants will be included in the analysis with the exclusion of the missing datapoint. If the overall proportion of missing questionnaire items was less than 5% and the participant had less than one missing item for every ten items, their mean at that specific time point was used to impute the missing data.

#### Sample size calculation

Mean effect sizes of current interventions on social functioning are around *d* = 0.5 [52]. With the G-power software package, the effect size is calculated based on an estimated effect size on the primary outcome of 0.5 [13, 53, 54], two groups, statistical power of 0.8, alpha of 0.05 and a standard deviation of 0.9. The required sample size of 102, with a correction for an estimated 13% [55] drop-out, amounts to *N* = 116.

### Data management

Participant data will be pseudonymized using a study ID. Data will be captured and stored in the Redcap and Roqua database system within the protected servers from the UMCG. Personal data such as phone numbers or (email) addresses to contact the participants during the research period will be strictly separated from other research data and is only accessible to the researcher and research assistant(s). The delegation log records which individual holds which role in the study. After completing the research project (i.e. data collection, data analysis, and publication of articles with primary and secondary outcomes), all research data will be transferred to the UMCG Research Drive for long-term storage. Research data will be stored for 15 years after the data collection has been completed (i.e. last research assessment for the last patient has been performed) in accordance with the NFU guidelines for WMO-compliant research. This data will be stored in a study-specific folder and is initially only accessible to the PI. Third parties can submit a research proposal and data request to the PI. The PI evaluates if the request falls within the scope of the research question and informed consent. More information regarding data management can be requested from the PI.

### Adverse events

The study adheres to the Medical Ethics Committee’s guidelines [56] regarding adverse events, and all participants are insured in the event of any trial-related harm.

## Discussion

The primary objective of this study is to evaluate the effect of a novel, modular VR intervention for improving social interaction difficulties in people with a psychotic disorder. It is hypothesized that the VR-SOAP group will show greater improvements in the quantity and quality of social contacts, leisure activities and social participation than the VRelax group at post-treatment and follow-up and we expect that these improvements are mediated by changes in underlying processes.

Although NICE guidelines consider social functioning as one of the “critical” outcome measures, current approaches have shown only moderate effects to this end [11–13]. Given that in the Netherlands alone around 100,000 people are receiving treatment in a mental health care setting for a psychotic disorder, many of whom have problems with social interactions [57], more effective interventions to improve social functioning are highly needed.

If VR-SOAP proves to be effective, it provides young adults with a psychotic disorder much-needed support to engage in social activities and to participate in society. Additionally, since the treatment consists of multiple modules targeting different transdiagnostic factors this trial might provide input for new treatments to improve social functioning in a range of symptoms and disorders, e.g. mood, autism spectrum and anxiety disorders.

## Data Availability

Data will be stored in the clinical data system “RedCap” provided by the Trial Coordination Center of the University Medical Center Groningen. A data request form will be made available. Third parties can submit a data request form to the investigator.

## Abbreviations

VR-SOAP: Virtual Reality Treatment for Social Activities and Participation
ESM: Electronic sampling method
TAU: Treatment as usual
SFS: Social Functioning Scale
SIG: Schaal Interpersoonlijk Gedrag
SERS-SF: Self-Esteem Rating Scale Short-Form
SIAS: Social Interaction Anxiety Scale
ISMI: Internalized Stigma of Mental Illness Scale
DAQ: Dating and Assertion Questionnaire
R-GPTS: Revised Green et al. Persecutory and Paranoid Thought Scale
BLERT: Bell-Lysaker Emotion Recognition Task
BNSS: Brief Negative Symptom Scale
PANSS: Positive and Negative Syndrome Scale
IPC: I-group Presence Questionnaire
